# New Biosensor for Determination of Neuropilin-1 with Detection by Surface Plasmon Resonance Imaging

**DOI:** 10.3390/s23084118

**Published:** 2023-04-19

**Authors:** Anna Sankiewicz, Beata Zelazowska-Rutkowska, Ewelina Gorska, Adam Hermanowicz, Ewa Gorodkiewicz

**Affiliations:** 1 Bioanalysis Laboratory, Faculty of Chemistry, University of Bialystok, Ciolkowskiego 1K, 15-245 Bialystok, Poland; ewka@uwb.edu.pl; 2 Department of Pediatric Laboratory Diagnostics, Medical University of Bialystok, Waszyngtona 17, 15-274 Bialystok, Poland; zelazowskab@wp.pl; 3 Independent Researcher, Stoleczna 7, 15-879 Bialystok, Poland; wiatrowa@wp.pl; 4 Department of Pediatric Surgery and Urology, Medical University of Bialystok, Waszyngtona 17, 15-274 Bialystok, Poland; ahermanowicz@wp.pl

**Keywords:** neuropilin-1, biosensor SPRi, serum, saliva

## Abstract

Neuropilin-1 is transmembrane protein with soluble isoforms. It plays a pivotal role in both physiological and pathological processes. NRP-1 is involved in the immune response, formation of neuronal circuits, angiogenesis, survival and migration of cells. The specific SPRI biosensor for the determination of neuropilin-1 was constructed using mouse monoclonal antibody that captures unbound NRP-1 form body fluids. The biosensor exhibits linearity of the analytical signal between 0.01 and 2.5 ng/mL, average precision value 4.7% and recovery between 97% and 104%. The detection limit is 0.011 ng/mL, and the limit of quantification is 0.038 ng/mL. The biosensor was validated by parallel determination of NRP-1 in serum and saliva samples using the ELISA test, with good agreement of the results.

## 1. Introduction

Neuropilins (NRP) are multifunctional receptors that were first identified because of their key role in mediating and directing the axons of the developing nervous system. Initially, neuropilins were described as semaphorin (SEMA) coreceptors [1,2]. Subsequently, Soker et al. identified the neuropilins as receptors for vascular endothelial growth factor (VEGF) [3]. There are two main isoforms in the neuropilin family: neuropilin-1 and neuropilin-2. NRP-1 and NRP-2 have a similar domain structure and an overlapping set of ligands [4]. Their amino acid sequences are 44% similar [5]. The primary structure of NRP-1 and NRP-2 consists of four domains: the CUB (a1/a2, homologous to complement components C1r/C1s) domain, the FV/FVIII (b1/b2, coagulation factor V/VIII homology) domain, the MAM domain (homologous to meprin, A5, μ), and a domain that contains a transmembrane helix and a short cytoplasmic tail. The soluble molecule of NRP consists of the same extracellular structure as the transmembrane NRP receptor, but is missing the transmembrane and the cytoplasmic part of the NRP-1 receptor [6]. The a and b domains bind particular endogenous ligands. The domains termed a1 and a2 are essential for binding to the SEMA domain of the semaphorin family. The b1 and b2 domains mediate binding to various isoforms of the vascular endothelial growth factor (VEGF) family. The MAM domain separates the b2 and transmembrane domains and mediates dimerization with other receptors. The cytoplasmic part contains no catalytic activity, but is the binding site of the PDZ (PSD-95/Dlg/ZO-1) domain of the protein interacting with NRP-1 (NIP—neuropilin interacting protein; or GIPCG—alpha interacting protein, C-terminus) [6,7]. Despite the similarities in the structure of neuropilins, there are differences between the ligands binding to NRP-1 and NRP-2. For example, VEGF-B, VEGF-E and SEMA-3A are bound by NRP-1 but do not interact with NPR-2. However, other ligands for NRPs have also been reported [7,8]. 

Neuropilins are transmembrane proteins that play a pivotal role in the control of neuronal guidance and VEGF signaling pathways, which underlies both physiological and pathological processes. Studies over the past years have revealed the involvement of neuropilins, especially NRP-1, in the immune response [9], angiogenesis [10], and survival and migration of cells [7,11]. NRP-1 is expressed in various tissues of the human body, especially in blood endothelial cells, vascular smooth muscle cells, retinal vasculature, neurons, and epithelial cells lining the respiratory and gastrointestinal tracts [12], as well as in immune cells, such as macrophages, dendritic cells (DCs), T cells, B cells, and mast cells [9]. 

The literature reveals great interest in neuropilin-1 in the context of the mediation of tumor development and progression, as its increased expression in the tumor vasculature has been observed. NRP-1 overexpression is seen in cancers of the pancreas, prostate, colorectal, breast, large intestine and kidneys, melanomas, glioblastoma, leukemias or lymphomas [13,14,15,16,17]. Because NRP-1 is involved in endothelial-dependent immune responses in the human brain, it may play a role in neurodegenerative diseases [18,19,20]. In the last two years, since the emergence of the SARS-CoV-2 pandemic, neuropilin-1 has been studied as an ally in the spread of the disease [12,18,21,22]. It has been observed that the presence of NRP-1 on the host cell surface facilitates the spread of SARS-CoV-2 infection, because the SARS-CoV-2 spike protein can bind to the b1,b2 domain of NRP-1 [12]. 

The determination of neuropilin-1 was formerly most often based on evaluation of its cell or tissue expression. For this purpose, flow cytometry [23] or Western blot [24] were used. However, detecting neuropilin-1 in tissue is invasive, complex and expensive compared with measuring the level of circulating NRP-1 in body fluids. In recent years, increasing numbers of studies on the detection of plasma NRP-1 levels have been published [15,25,26,27]. There are also reports of NRP-1 determination in urine [28]. For the detection of NRP-1 in body fluids, the enzyme-linked immunosorbent assay (ELISA) has been used. 

Biosensors are often a good alternative method of biomolecule detection. They are used in various fields of medicine as they enable the fast detection of the initiation of disease and monitoring of its progression. The phenomenon of surface plasmon resonance (SPR) is used as a detection method in biosensors designed to detect potential biomarkers. The SPR phenomenon occurs in the surface layers of metal. Gold is the metal most often used as the basic layer of the biosensor. The measurement is based on the Kretschmann configuration [29]. When polarized light strikes a metal film at the interface of media with different refractive indices, it excites oscillations of surface free electrons (known as surface plasmons). This phenomenon is sensitive to any changes on the metal surface. The beam of light has a certain wavelength and falls at a certain angle. When subsequent layers are applied to the metal, the angle that causes the SPR effect changes and it is seen as a dip in the intensity of the reflected light at the SPR angle. The SPR measurements as applied in biosensors are used to monitor the changes in refractive index near the surface of the metal due to the binding of molecules to the surface. The SPR technique enables real-time measurement with high sensitivity and without the need for labels. Surface Plasmon Resonance Imaging (SPRi) is one of the variants of the SPR technique, and it combines its advantages with multiarray measurement. In this variant, measurements are taken at a constant wavelength and angle. The change in reflected light is measured using images from the CCD camera. To improve the detection performance of the SPR and the sensitivity of the method, efforts were made to test various modifications of the SPR technique by using various materials and nanostructures [30,31,32].

However, due to the simplicity of the SPRI biosensor construction, the technique still remains attractive for development of new biosensors.

In this work, we introduce a new method of neuropilin-1 determination based on an SPRi biosensor. This is the first marker-free biosensor to be applied in multiarray detection of free neuropilin-1 in body fluids. The mouse monoclonal antibody specific for neuropilin-1 was selected as the recognition element of the biosensor. The aim of the experiments was to optimize the analytical parameters of method and to validate it by measuring NRP-1 concentration in biological samples. For comparative evaluation of the results obtained with the developed method, an ELISA test was performed on the same samples. 

## 2. Materials and Methods

### 2.1. Materials and Reagents 

The recombinant human neuropilin-1, neuropilin-2, mouse monoclonal antibody of human neuropilin-1 (R&D Systems, Minneapolis, MN, USA), vascular endothelial growth factor VEGFA (ABCAM, Cambridge, UK), cysteamine hydrochloride, N-ethyl-N′-(3-dimethylaminopropyl) carbodiimide (EDC), N-hydroxysuccinimide (NHS) (ALDRICH, Munich, Germany), absolute ethanol, acetic acid, hydrochloric acid, sodium hydroxide, sodium chloride, sodium carbonate, sodium acetate (POCh, Gliwice, Poland), HBS-ES buffer pH = 7.4 (0.01 M HEPES, 0.15 M sodium chloride, 0.005% Tween 20, 3 mM EDTA), Phosphate Buffered Saline (PBS) pH = 7.4, and carbonate buffer pH = 8.5 (BIOMED, Lublin, Poland) were used. Argon N 5.0 with Ar ≥ 99.999% content was used (AIR LIQUIDE Polska Sp.z o.o., Kraków, Poland). The bases of the immunosensors were chips covered with a layer of gold (Ssens, Uithoorn, The Netherlands).

### 2.2. Biological Materials

The biological materials used in the research were:

– blood plasma samples from patients with diagnosed multiple sclerosis and from smokers (Podlasie Psychogeriatric Center in Bialystok and the Department of Neurology, Medical University of Białystok, Poland);
– saliva samples from patients before endodontic treatment and from healthy volunteers (Ortho-Dent and Dentalblue, Bialystok, Poland).

The study obtained the consent of the relevant bioethical committee (license No. R-I-002/41/2019). The tested samples were diluted in a PBS solution so that the range of signals received from the detector lay within the range of the calibration curves. 

### 2.3. Instrumentation

SPRi experiments were performed using a stationary device developed by our laboratory. The details of the construction are described in the previous paper [33]. The main parts of the SPRi apparatus used were a He-Ne laser, two glass lenses L1 (f 3 mm) and L2 (f 300 mm), two polarizers (P1 and P2), a mirror, a glass prism, and a CCD camera. The biosensor was placed on a prism and the Kretschmann configuration was used for measurements. The SPRi signal was calculated from the pictures taken with the CCD camera using ImageJ software (NIH, version 1.32). 

The Anthos ELISA Reader (Salzburg, Austria) was used for the ELISA tests. The quantitation was performed using Human Neuropilin-1 ELISA Kit according to the recommendations of the manufacturer’s protocol.

A Quartz Crystal Microbalance (QCM, MethromAutolab B.V., Utrecht, The Netherlands) connected to a potentiostat/galvanostat (MethromAutolab B.V. PGSTAT 302N) was used to confirm the formation of successive immunosensor layers. 

### 2.4. Procedures

#### 2.4.1. Sensor Preparation

**Figure 1:**
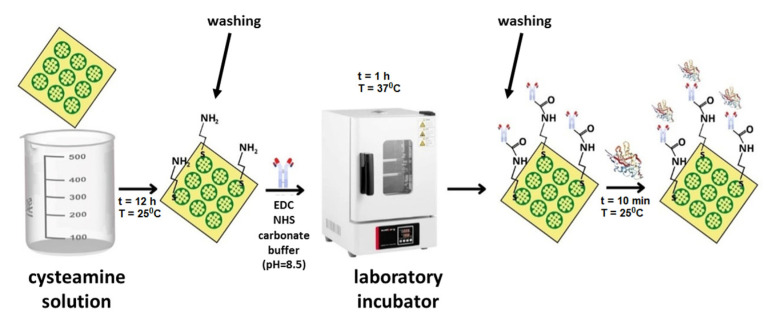
Scheme of the sensor preparation procedure.

The biosensors were based on glass chips with sputtered gold, purchased directly from the manufacturer (SSens, Uithoorn, The Netherlands). The preparation of the biosensor began with the immobilization of a linker layer to enable binding of the receptor (antibody). The gold plate was immersed in a 20 mM alcoholic cysteamine solution for a minimum of 12 h at room temperature. After the required time, the plate was rinsed in anhydrous ethyl alcohol and water. The next step was the binding of NRP-1 antibody. For this purpose, 2–3 µL of antibody solution of the appropriate concentration was applied to the cysteamine layer, and the chip prepared in this way was placed in an incubator for 1 h at 37 °C. After this time, the biosensor was washed with HBS-ES solution and distilled water to remove excess unbound antibodies. To eliminate non-specific adsorption, a BSA solution (*C* = 1 mg/mL) was applied to the biosensor surface and rinsed with distilled water. The biosensor prepared in this way enabled the capture of NRP-1 from the solution. About 2–3 µL of the analyzed solution was dropped onto the active sites and left for 10 min. The biosensor was then rinsed with HBS-ES solution and water. All measurements were carried out at pH = 7.40 (based on information from the safety data sheets of the reagents). The procedure for the preparation of the sensor is shown schematically in Figure 1. 

#### 2.4.2. SPRi Measurement

The prepared biosensor was placed on a prism in the SPRi device, and the appropriate angle of incidence of the laser light at which the measurements were to be conducted was set. Using a CCD camera, photographs were taken at a fixed SPR angle. First, an image of the antibody layer was recorded. Next, the interaction of the antibody with the solution containing neuropilin-1 was performed. After rinsing and drying of the biosensor surface, a second image was recorded. As a result of the binding of the analyte to the receptor, there was a change in the refractive index (RI), which was detected as changes in the reflected light intensity recorded on the CCD camera image. Mathematical 2D image analysis was performed using ImageJ software (NIH, Version 1.8.0_172). The final SPRi signal was calculated based on the difference of the signals obtained before and after the interaction of the antibody with the analyte.

#### 2.4.3. QCM Measurements

A quartz crystal microbalance (QCM) was used to check the formation of molecular complexes on the surface of the biosensor. The crystal placed in the measuring cell (3 mL) had a resonance frequency of 6 MHz and an area of 0.361 cm^2^. The gold layer was 100 nm thick. The procedure for creating successive layers on gold was similar to that used for the SPRi measurements. The first step was to pour 2 mL water into the measuring cell and to measure the oscillation changes (Δ*f*) of the quartz crystal with a gold layer. The water was then removed from the measuring cell and the crystal was dried with argon. Next, 2 mL cysteamine solution (20 mM) was poured in. The solution was left for 2 h to coat the gold with a layer of cysteamine. After this time, the cysteamine solution was removed and the crystal was rinsed with anhydrous ethyl alcohol and water, and dried with an argon stream. The oscillation changes for the cysteamine layer were then measured. Next, the antibody solution (activated with the EDC-NHS mixture in carbonate buffer) was placed in the measuring cell and left for 1 h to immobilize it on the cysteamine layer. The Δ*f* value for the antibody layer was measured. In the next step, the antibody–antigen reaction was performed. For this purpose, the neuropilin-1 solution was placed in the microbalance cell for 10 min, and then Δ*f* was measured again. The QCM analysis was supported by dedicated NOVA 2.1 software.

#### 2.4.4. ELISA—Measurements

The determination of neuropilin-1 concentration was performed using Human Neuropilin-1 Quantikine ELISA Kit (R&D Systems, Minneapolis, MN, USA) according to the manufacturer’s instructions.

## 3. Results

### 3.1. Optimization of Conditions for NRP-1 Determination 

**Figure 2:**
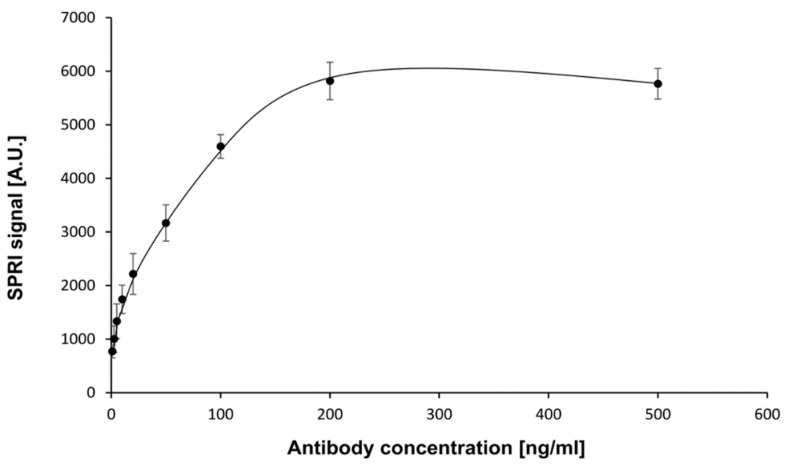
Dependence of SPRI signal (Arbitrary Units) on mouse NRP-1 antibody concentration. NRP-1 concentration: 5 ng/mL. pH = 7.4. Error bars were calculated for 12 independent measurements for each concentration at a 95%confidence level.

The first stage in the development of the biosensor was the selection of an appropriate capture element (receptor) and determination of its optimal concentration. Mouse monoclonal antibody of human neuropilin-1 was chosen as a receptor. Experiments were performed at pH = 7.4. Antibody solutions were prepared in PBS buffer with concentrations in the range of 1.00–500 ng/mL. The cysteamine-coated chip was treated with the antibody solution by placing drops with successive concentrations of antibody on the active sites. After immobilization of the antibody, the biosensor was treated with neuropilin-1 solution at a constant NRP-1 concentration (5 ng/mL). The measurements were carried out according to the procedure described in Section 2.4.1 and Section 2.4.2. The measurement results are presented in the form of a plot of the SPRi signal versus antibody concentration (Figure 2).

The curve obtained was of the Langmuir isotherm type. The amount of immobilized antibody was related to the antibody concentration. The SPRi signal increased until saturation was reached. A concentration of antibody of 200 ng/mL and above provided the highest specific signal and was therefore used in the subsequent stages of the study. 

### 3.2. Establishment of the Standard Curve

**Figure 3:**
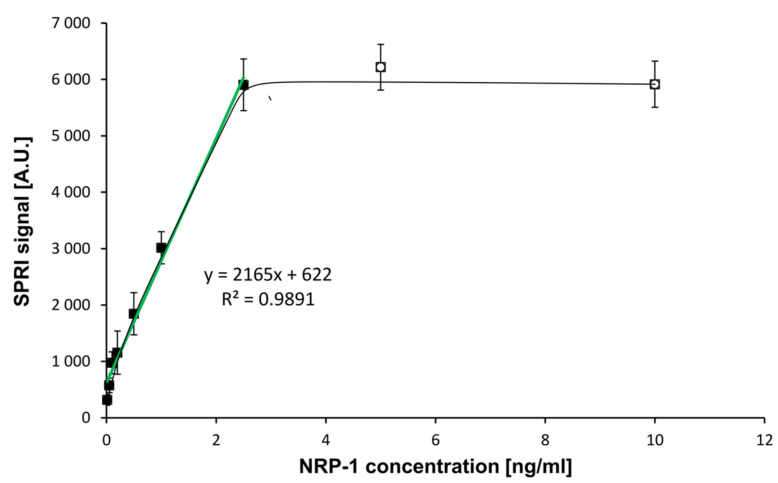
Dependence of the SPRI signal (Arbitrary Units) on NRP-1 concentration. (**―**): in the full range of tested concentrations, (**―**): calibration curve. NRP-1 antibody concentration: 200 ng/mL, pH = 7.4. Error bars were calculated for 12 independent measurements for each concentration at a 95% confidence level.

Instrument calibration and establishment of the standard curve is an essential stage in quantitative measurement procedures. To establish the relationship between the instrument’s response and NRP-1 concentration, a set of nine standard solutions containing a known amount of the analyte was prepared. The concentration of NRP-1 was in the range of 0.01–10.0 ng/mL. The calibration standard of NRP-1 was diluted with PBS solution. Experiments were performed at an antibody concentration of 200 ng/mL. The effect of neuropilin-1 concentration on the SPRi signal (the calibration curve) is shown in Figure 3.

A curve of Langmuirian shape was obtained. Saturation of the biosensor surface was observed at NRP-1 concentrations above 2.50 ng/mL. The limit of detection (LOD) was calculated by the formula 3.3 SD/A, where A was the slope of the calibration curve. The limit of quantification LOQ was calculated as 10 SD/A. The results obtained and the parameters of the ELISA test are presented in Table 1.

### 3.3. Recovery and Precision of the SPRi Method for Determination of Neuropilin-1

The precision of the developed SPRi biosensor was determined by applying five standard solutions with appropriate concentrations of the reference material (0.05, 0.5, 1.00, 2.00 and 2.50 ng/mL) to the active sites of the biosensors. These concentrations are within the linear range of the calibration curve. For each sample, 12 independent measurements were conduced. The percentage recovery of the added NRP-1 and RSD were calculated for each of the samples (Table 2).

The results showed percentage recoveries at all concentration levels in the range of 97–106%, while CV values were in the range of 3.4–5.8%. The results were within the accepted limits, namely from 80.00% to 120.00% and not more than 20.0% [34], which indicates that satisfactory precision and recovery were achieved.

### 3.4. Selectivity of Biosensor

Selectivity is the ability of an analytical method to differentiate and quantify the analyte in the presence of other components in the sample. The interaction between the biorecognition element and target bioanalyte should be based on a strong and selective mutual affinity. In our experiment, the mouse monoclonal antibody of human neuropilin-1 was used as the biorecognition element. According to the manufacturer, this antibody detects human neuropilin-1 directly and it does not cross-react with recombinant human neuropilin-2. The selectivity of the developed method was evaluated by the measurement of NRP-1 concentration in the presence of neuropilin-2 and other interferents such as VEGF-A and albumin. Neuropilin-2 was selected as a potential interferent due to the similar structure of its domains to those of NRP-1. The second potential interferent was VEGF-A, which binds to NRP-1 with high affinity. The third interferent studied, albumin, constitutes about 60% of all proteins and is capable of binding and transporting various substances. It was investigated to exclude potential nonspecific adsorption on the surface of the chip’s active sites.

**Figure 4:**
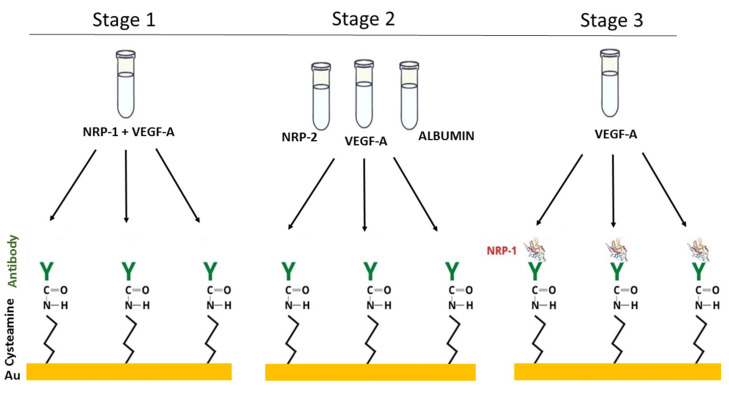
The experiment to test the selectivity of the biosensor.

The test of the biosensor’s selectivity was carried out in three stages. In the first stage, mixtures of neuropilin-1 with VEGF-A were prepared in various ratios (c_NRP1_:c_VEGFA_ = 1:10, 1:1, 10:1, 100:1). These mixtures were dropped onto the active sites. In the second step, the antibody’s selectivity was studied. A solution of NRP-2, VEGF-A and albumin was applied to the antibody. The concentrations of NRP-2 were 1.00 and 10.0 ng/mL, those of VEGF-A were 0.01, 0.10, 1.00 and 10.0 ng/mL, and those of albumin were 1.00, 10.0 and 100 ng/mL. The third step was to check whether the antibody-bound neuropilin-1 captures VEGF-A from the biological material. For this purpose, solutions of NRP-1 at a concentration of 1 ng/mL were applied to the antibody and left for 10 min. After washing with water and removal of unbound NRP-1 molecules, VEGF-A solutions with concentrations of 0.01, 0.10, 1.00 and 10.0 ng/mL were dropped onto each of the sites. The experimental procedure is illustrated schematically in Figure 4.

The results of the selectivity tests are presented in Table 3. They show that the antibody used does not capture NRP-2, VEGF-A or albumin. The NRP-1 associated with the antibody does not interact with VEGF-A (recoveries of 103–107%). The addition of VEGF-A in excess to the standard solution of neuropilin-1 resulted in its complexing, and the recovery was at levels of <1%. When the ratio of concentrations of NRP-1 and VEGF-A was 1:1, the recovery was 36%. In the case of an excess of NPR-1 over VEGF-A (10:1 and 100:1), the recovery was 85% and 98%, respectively. The concentration of VEGF-A in plasma is at least 100 times lower than that of NRP-1 [15,35], and therefore it should not interfere with the quantification of the test protein. It can be concluded from our study that the presented biosensor detects unbound neuropilin-1.

### 3.5. QCM Studies of the Biosensor Surface

**Figure 5:**
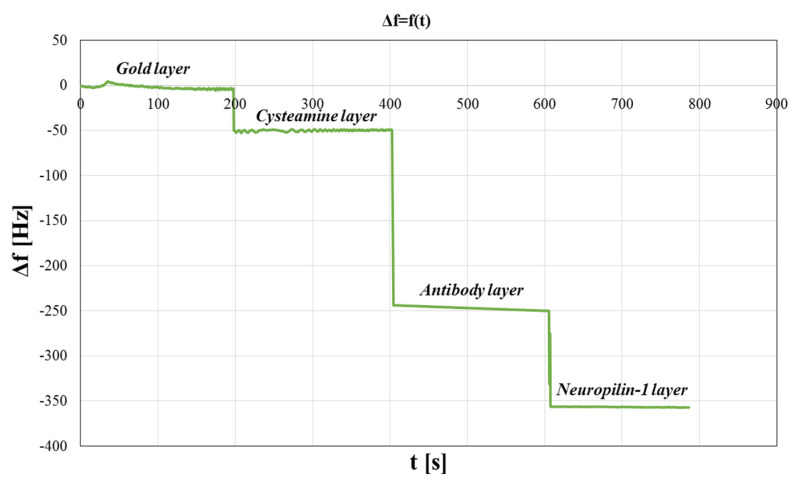
QCM studies of the biosensors surface. Variation of frequency as a function of time.

The QCM is a mass-sensitive transducer and can detect the adsorption of biochemical species on the sensor surface. Therefore, QCM was used to test the formation of successive layers of the biosensor. The results are presented in Figure 5. Frequency changes Δ*f* (Hz) as a function of time *t* (s) were observed, and confirmed the formation of successive layers of the sensor. 

### 3.6. Determination of NRP-1 in Natural Samples 

**Figure 6:**
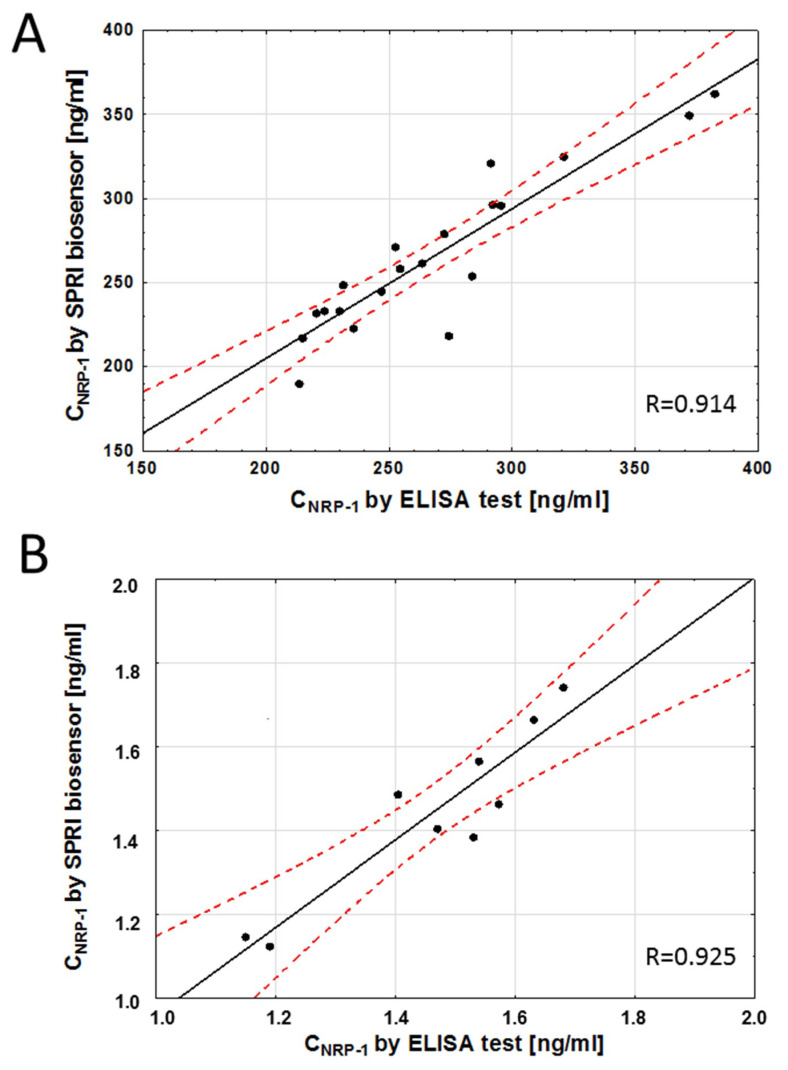
Correlation between serum (**A**) and saliva (**B**) NRP-1 levels quantified with the SPRI-biosensor and the traditional ELISA assay. Each point represents a single patient. The plot of correlation (**―**), confidence interval ( - - - ).

The SPRi biosensor was tested to detect NRP-1 in serum and saliva samples. The accuracy of the developed method was validated with the ELISA method. NRP-1 was quantified in the same samples using both methods, and the detected concentrations were compared. The results are presented in Figure 6. The Pearson correlation coefficient was calculated to assess the correlation between the concentration values obtained with both methods, and it was 0.914 (*p* < 0.05) for the serum samples and 0.925 (*p* < 0.05) for the saliva samples. These results indicate that the NRP-1 concentration values obtained with the two tests are very similar. Thus, the developed SPRi biosensor can be used to quantify neuropilin-1 in both serum and saliva. The neuropilin-1 concentration range obtained was 190–362 ng/mL in serum and 1.13–1.74 ng/mL in saliva. 

## 4. Discussion 

There are few reports in the literature on methods of neuropilin-1 quantification. ELISA tests are most commonly used. In this study, a biosensor was developed to determine neuropilin-1 concentration by the SPRi technique. The sensor operates on the basis of an interaction between a specific antibody as recognition element and NRP-1 in solution. The biosensor exhibited linearity of the analytical signal response and acceptable precision and recovery. In the developed method, a lower LOQ was obtained compared with the commercial ELISA test (Table 1). The results of selectivity tests showed that the antibody used captures only neuropilin-1 not bound to VEGF-A. Gagnon et al. studied the binding of recombinant soluble NRP-1 to VEGF-A165. The analysis showed that sNRP-1 binds to VEGF-A165 with a K_d_ value of around 5–10 × 10^−9^ M [36]. Our study showed that VEGF-A used as an interfering biomolecule binds NRP-1, and at a 1:1 concentration ratio of NRP-1 and VEGF-A, the recovery rate was only 36%. The concentration of VEGF-A in serum was found to be in the range of 133–1657 pg/mL for control subjects and patients with ischemic and hemorrhagic strokes [30], and mean serum values of sNRP-1 were reported by Rachner at 1.88  ±  0.52 nmol/L (= 130.83  ±  36.24 ng/mL) for patients with breast cancer [15]. The range of NRP-1 concentrations is therefore 1000 times greater than that of VEGF, and thus the latter should not interfere with the determination of the amount of free neuropilin-1. The concentrations in serum obtained in our studies range from 190 to 362 ng/mL. This was the first time that a biosensor was used to determine the concentration of NRP-1 in saliva. The concentration of sNRP-1 in gingival crevicular fluid (GCF) samples from periodontitis patients was determined [37]. This study discovered a good correlation between the results obtained from the SPRi and the ELISA measurements of NRP-1 levels. The correlation coefficient for the quantitative determinations of NRP-1 concentration in serum and saliva was above 0.9. The deviations from the regression line are probably due to experimental error in either the SPRi biosensor or the ELISA immunoassay, or both. The complexity of the natural samples or the use of different antibodies in the two assays may also have contributed to the deviations. 

One of the most important characteristics distinguishing the SPRi biosensor from other techniques such as ELISA is that it does not require the use of any labels that may affect the natural functionality of the biomolecules. SPRi biosensors, therefore, also offer economic advantages, as they save the cost of the expensive labeling reagents used. The volumes of the samples and reagents used are of the order of 2–5 µL. This factor also reduces waste. The SPRi sensor chip used in our experiment can be made reusable by a regeneration mixture [38]. 

Like any method, the SPRi biosensor also has its limitations. The current limits in SPR technique are predominantly determined by properties of the surface plasmon phenomenon and the present state of development of optical technology. In imaging version of SPR, the limitation is explained by the use of camera images for measurements. The sensitivity of measurements depends on picture resolution. It is therefore essential to use detectors with a high signal-to-noise ratio. To improve the sensitivity of the SPR sensing, the various materials and nanostructures to surface modification can be used [32]. 

Influence of the interference of non-specific binding of other molecules on the outcome signals is also a disadvantage of SPRI sensing. However, this can be overcome by using a highly selective receptor, blocking the surface layer or appropriate dilution of the sample.

Due to the important role of neuropilin-1 in immune processes, cancer and neurodegenerative disorders, the developed method may serve as a tool to study its role as a potential biomarker of diseases. With NRP-1 emerging as a promising therapeutic target for diseases [9,12,13], it is necessary to study the level of NRP-1 and the mechanisms of action of this protein in various signaling pathways in the body. 

## 5. Conclusions

In summary, we have described a new method for detecting the soluble form of NRP-1 in serum and saliva. A specific SPRi biosensor for the determination of neuropilin-1 was constructed using mouse monoclonal antibody, which captures unbound NRP-1 from body fluids. The developed sensor displayed good sensitivity and precision, as well as acceptable values of recovery. The results obtained with the SPRi biosensor are similar to those obtained by the commercial ELISA test. The quantification of NRP-1 in serum or saliva can serve to study NRP-1 as a potential biomarker for more personalized diagnostic and treatment plans in cases of various diseases. 

## Figures and Tables

**Table 1:** Parameters of the calibration curve obtained by the SPRi biosensor and the ELISA method.

Method	Linear Dynamic Range	R^2^	LOD	LOQ
SPRI biosensor	0.01–2.50 ng/mL	0.9891	0.011 ng/mL	0.032 ng/mL
ELISA	78.1–5000 pg/mL	0.9794	n/d	9.330 pg/mL

**Table 2:** Recovery and precision of the developed method.

Series	Spiked (ng/mL)	Found (ng/mL)	SD (ng/mL)	CV (%)	Recovery (%)
1	0.05	0.052	0.003	5.8	104
2	0.50	0.517	0.025	4.8	103
3	1.00	0.965	0.044	4.6	97
4	2.00	1.955	0.097	4.7	98
5	2.50	2.439	0.084	3.4	98

**Table 3:** Analytical specificity of the developed biosensors.

	C_interferent_ (ng/mL)	Added C_NRP-1_ (ng/mL)	Found C_NRP-1_ (ng/mL)	Recovery (%)
**STAGE1**	**Mixture NRP1 + VEGF-A**			
	0.01	1	0.98	98
	0.10		0.85	85
	1.00		0.36	36
	10.0		<0.01	<1
**STAGE 2**	**NRP-2**			
	1.00	not added	not found	0
	10.0			0
	**VEGF-A**			
	0.01	not added	not found	0
	0.10			0
	1.00			0
	10.0			0
	**Albumin**			
	1.00	not added	not found	0
	10.0			0
	100			0
**STAGE 3**	**VEGF-A**			
	0.01	1	1.04	103
	0.10		1.06	106
	1.00		1.07	107
	10.0		1.05	105

## Data Availability

Not applicable.

## References

[1] Kolodkin A.L., Levengood D.V., Rowe E.G., Tai Y.T., Giger R.J., Ginty D.D. (1997). Neuropilin is a semaphorin III receptor. Cell.

[2] He Z., Tessier-Lavigne M. (1997). Neuropilin is a receptor for the axonal chemorepellent semaphorin III. Cell.

[3] Soker S., Takashima S., Miao H.Q., Neufeld G., Klagsbrun M. (1998). Neuropilin-1 is expressed by endothelial and tumor cells as an isoform- specific receptor for vascular endothelial growth factor. Cell.

[4] Parker M.W., Guo H.F., Li X., Linkugel A.D., Vander Kooi C.W. (2012). Function of members of the neuropilin family as essential pleiotropic cell surface receptors. Biochemistry.

[5] Broz M., Kolarič A., Jukič M., Bren U. (2022). Neuropilin (NRPs) Related Pathological Conditions and Their Modulators. Int. J. Mol. Sci..

[6] Pellet-Many C., Frankel P., Jia H., Zachary I. (2008). Neuropilins: Structure, function and role in disease. Biochem. J..

[7] Sulpice E., Plouët J., Bergé M., Allanic D., Tobelem G., Merkulova-Rainon T. (2008). Neuropilin-1 and neuropilin-2 act as coreceptors, potentiating proangiogenic activity. Blood.

[8] Wild J.R.L., Staton C.A., Chapple K., Corfe B.M. (2012). Neuropilins: Expression and roles in the epithelium. Int. J. Exp. Pathol..

[9] Roy S., Bag A.K., Singh R.K., Talmadge J.E., Batra S.K., Datta K. (2017). Multifaceted role of neuropilins in the immune system: Potential targets for immunotherapy. Front. Immunol..

[10] Benwell C.J., Johnson R.T., Taylor J.A.G.E., Price C.A., Robinson S.D. (2022). Endothelial VEGFR Coreceptors Neuropilin-1 and Neuropilin-2 Are Essential for Tumor Angiogenesis. Cancer Res. Commun..

[11] Lyu Z., Jin H., Yan Z., Hu K., Jiang H., Peng H., Zhuo H. (2020). Effects of nrp1 on angiogenesis and vascular maturity in endothelial cells are dependent on the expression of sema4d. Int. J. Mol. Med..

[12] Abebe E.C., Ayele T.M., Muche Z.T., Dejenie T.A. (2021). Neuropilin 1: A novel entry factor for SARS-CoV-2 infection and a potential therapeutic target. Biol. Targets Ther..

[13] Grandclement C., Borg C. (2011). Neuropilins: A new target for cancer therapy. Cancers.

[14] Jubb A.M., Strickland L.A., Liu S.D., Mak J., Schmidt M., Koeppen H. (2012). Neuropilin-1 expression in cancer and development. J. Pathol..

[15] Rachner T.D., Kasimir-Bauer S., Goebel A., Erdmann K., Hoffmann O., Rauner M., Hofbauer L.C., Kimmig R., Bittner A.K. (2021). Soluble Neuropilin-1 is an independent marker of poor prognosis in early breast cancer. J. Cancer Res. Clin. Oncol..

[16] Jin Q., Ren Q., Chang X., Yu H., Jin X., Lu X., He N., Wang G. (2021). Neuropilin-1 predicts poor prognosis and promotes tumor metastasis through epithelial-mesenchymal transition in gastric cancer. J. Cancer.

[17] Chaudhary B., Elkord E. (2015). Novel expression of Neuropilin 1 on human tumor-infiltrating lymphocytes in colorectal cancer liver metastases. Expert Opin. Ther. Targets.

[18] Zalpoor H., Akbari A., Samei A., Forghaniesfidvajani R., Kamali M., Afzalnia A., Manshouri S., Heidari F., Pornour M., Khoshmirsafa M. (2022). The roles of Eph receptors, neuropilin-1, P2X7, and CD147 in COVID-19-associated neurodegenerative diseases: Inflammasome and JaK inhibitors as potential promising therapies. Cell. Mol. Biol. Lett..

[19] Daneshvar Kakhaki R., Kouchaki E., Dadgostar E., Behnam M., Tamtaji O.R., Nikoueinejad H., Akbari H. (2020). The correlation of helios and neuropilin-1 frequencies with parkinson disease severity. Clin. Neurol. Neurosurg..

[20] Wang Y., Cao Y., Mangalam A.K., Guo Y., LaFrance-Corey R.G., Gamez J.D., Atanga P.A., Clarkson B.D., Zhang Y., Wang E. (2016). Neuropilin-1 modulates interferon-γ-stimulated signaling in brain microvascular endothelial cells. J. Cell Sci..

[21] Chapoval S.P., Keegan A.D. (2021). Perspectives and potential approaches for targeting neuropilin 1 in SARS-CoV-2 infection. Mol. Med..

[22] Gudowska-sawczuk M. (2021). The Role of Neuropilin-1 (NRP-1) in SARS-CoV-2 Infection: Review. J. Clin. Med..

[23] Karczmarczyk A., Bilska S., Korpysz M., Purkot J., GrzĄŚko N., Hus M., Giannopoulos K. (2020). Expression and Clinical Significance of Neuropilin-1 in Patients With Multiple Myeloma. Anticancer Res..

[24] Ruffini F., D’Atri S., Lacal M.P. (2013). Neuropilin-1 expression promotes invasiveness of melanoma cells through vascular endothelial growth factor receptor-2-dependent and -independent mechanisms. Int. J. Oncol..

[25] Rzepakowska A., Żurek M., Grzybowski J., Kotula I., Pihowicz P., Górnicka B., Demkow U., Niemczyk K. (2020). Serum and tissue expression of neuropilin 1 in precancerous and malignant vocal fold lesions. PLoS ONE.

[26] Lu Y., Xiang H., Liu P., Tong R.R., Watts R.J., Koch A.W., Sandoval W.N., Damico L.A., Wai L.W., Meng Y.G. (2009). Identification of circulating neuropilin-1 and dose-dependent elevation following anti-neuropilin-1 antibody administration. MAbs.

[27] Gadermaier E., Tesarz M., Wallwitz J., Berg G., Himmler G. (2019). Characterization of a sandwich ELISA for quantification of total human soluble neuropilin-1. J. Clin. Lab. Anal..

[28] Torres-Salido M.T., Sanchis M., Solé C., Moliné T., Vidal M., Vidal X., Solà A., Hotter G., Ordi-Ros J., Cortés-Hernández J. (2019). Urinary neuropilin-1: A predictive biomarker for renal outcome in lupus nephritis. Int. J. Mol. Sci..

[29] Šípová H., Homola J. (2013). Surface plasmon resonance sensing of nucleic acids: A review. Anal. Chim. Acta.

[30] Garoli D., Calandrini E., Giovannini G., Hubarevich A., Caligiuri V., De Angelis F. (2019). Nanoporous gold metamaterials for high sensitivity plasmonic sensing. Nanoscale Horiz..

[31] Sreekanth K.V., Alapan Y., Elkabbash M., Ilker E. (2016). Health Research Alliance. Nat. Mater..

[32] Altug H., Oh S.H., Maier S.A., Homola J. (2022). Advances and applications of nanophotonic biosensors. Nat. Nanotechnol..

[33] Gorodkiewicz E., Sankiewicz A., Laudański P. (2014). Surface plasmon resonance imaging biosensors for aromatase based on a potent inhibitor and a specific antibody: Sensor development and application for biological material. Cent. Eur. J. Chem..

[34] United Nations Office on Drugs and Crime (2009). A Commitment to Quality and Continuous Improvement.

[35] Babkina A.S., Yadgarov M.Y., Ostrova I.V., Zakharchenko V.E., Kuzovlev A.N., Grechko A.V., Lyubomudrov M.A., Golubev A.M. (2022). Serum Levels of VEGF-A and Its Receptors in Patients in Different Phases of Hemorrhagic and Ischemic Strokes. Curr. Issues Mol. Biol..

[36] Gagnon M.L., Bielenberg D.R., Gechtman Z., Miao H.Q., Takashima S., Soker S., Klagsbrun M. (2000). Identification of a natural soluble neuropilin-1 that binds vascular endothelial growth factor: In vivo expression and antitumor activity. Proc. Natl. Acad. Sci. USA.

[37] Prieto D., Maurer G., Sáez M., Cáceres F., Pino-Lagos K., Chaparro A. (2021). Soluble Neuropilin-1 in gingival crevicular fluid from periodontitis patients: An exploratory cross-sectional study. J. Oral Biol. Craniofacial Res..

[38] Sankiewicz A., Tokarzewicz A., Gorodkiewicz E. (2015). Regeneration of surface plasmone resonance chips for multiple use Regeneration of surface plasmone resonance chips for multiple use. Bulgar. Chem. Commun..

